# The importance of sleep patterns in the incidence of coronary heart disease: a 6-year prospective study in Mashhad, Iran

**DOI:** 10.1038/s41598-023-29451-w

**Published:** 2023-02-18

**Authors:** Fatemeh Sadabadi, Susan Darroudi, Habibollah Esmaily, Zahra Asadi, Gordon A. Ferns, Amir Hooshang Mohammadpour, Amir Hossein Nooriyan, Majid Ghayour-Mobarhan, Mohsen Moohebati

**Affiliations:** 1 International UNESCO Center for Health-Related Basic Sciences and Human Nutrition, Mashhad University of Medical Sciences, Mashhad, Iran; 2 Social Determinants of Health Research Center, Mashhad University of Medical Sciences, Mashhad, Iran; 3 Division of Medical Education, Falmer, Brighton & Sussex Medical School, Brighton, BN1 9PH Sussex UK; 4 Pharmaceutical Research Center, Pharmaceutical Institute Technology, Mashhad University of Medical Sciences, Mashhad, Iran; 5 Department of Clinical Pharmacy, School of Pharmacy, Mashhad University of Medical Sciences, Mashhad, Iran; 6 Islamic Azad University of Mashhad (IAUM), Mashhad, Iran; 7 School of Medicine, Metabolic Syndrome Research Center, Mashhad University of Medical Sciences, Mashhad, 99199-91766 Iran; 8 Cardiovascular Research Center, School of Medicine, Mashhad University of Medical Sciences, Mashhad, Iran

**Keywords:** Cardiology, Medical research, Risk factors

## Abstract

Chronic shortened sleep can increase several cardiovascular risk factors, including depression, anxiety, metabolic syndrome, diabetes and hypertension. In the current study, we aimed to investigate the relationship between sleep patterns and the incidence of coronary heart disease (CHD). A total of 9704 healthy participants were recruited for the MASHAD cohort study. Within 6 years of follow-up, participants were categorized into four groups based on their number of hours of nightly sleep. Cox’s proportional hazard model was used to assess relative risks (RRs) and 95% confidence intervals (CIs). During the study, 235 heart problems, including myocardial infarction, stable angina and unstable angina, were confirmed. There were significant differences between men and women who had short and long nightly sleep (p < 0.05). The incidence of CHD was significantly higher in participants with very short night sleep durations than in those with longer hours of night sleep. The subjects with very short nightly sleep were more susceptible to unstable angina (RR: 2.614 (CI 1.354–5.047)) (p < 0.05). We found that shortened nightly sleep was associated with an increased incidence of coronary heart disease in an Iranian population. These findings suggest that sleep disorders, especially shortened night sleep, can be a risk factor for CHD.

## Introduction

Sleep is an essential indicator of well-being and health in humans. It has been proposed that a healthy sleep pattern is associated with reduced risks of different life-threatening diseases such as cardiovascular disease (CVD)^1,2^. Lack of or shortened sleep adversely affects physical health, temper, mood, and cognitive function^3^. People who complain of chronic loss and shortened sleep have problems staying asleep as long as qualified and generally suffer from sleepiness, being tired and lethargic, irritability, distressed and poor mood during the day^4,5^, so their quality of life is affected^3^.

Some studies indicated an emerging decrease in sleep duration in developed and industrialized countries such as the north American and European countries^5^. A study of more than 50 epidemiological studies showed that the prevalence of poor sleep and insomnia differs from 6 to 33%^6^. It is estimated that 10% to 30% of the US general population suffers from this problem^6^. Other investigations appraised that more than 30% of adults, as well as over 50% of the population older than 65 years, have current features of sleep disorders^5^.

In recent years, the problem of chronic loss or shortened sleep as well as insomnia has received special attention because some growing evidence suggests that poor sleep can increase some cardiovascular risk factors, such as the development of diabetes^3^, high blood pressure^7^, weight gain and obesity^8^, elevated resting heart rate (RHR)^4^, metabolic syndrome^9^, depression^10^, and immune system and cytokine titers^3^. Consequently, sleep seems to play important roles in cardiovascular health^8^. Coronary heart disease (CHD) is among the most important causes of fatality and morbidity worldwide^11,12^. The immune system, endocrine function, and inflammatory markers and cytokines seem to be associated with sleep duration and CVD^13^. However, to date, the available documents are not quite consistent, and most evidence has not been repeated clearly^14^.

In this study, we aimed to investigate the importance of pattern and timing of sleep in the incidence of coronary heart disease, a main cause of death worldwide, and its risk factors in a representative Iranian population sample from northeastern Iran after 6 years of follow-up.

## Materials and methods

### Study population

The MASHAD study, a cardiovascular risk factor cohort, was initiated in 2010 using a cluster-randomized methodology and invited volunteers aged 35–65 years who were living in the city of Mashhad in northeastern Iran^15^. Informed consent was obtained from all subjects. The exclusion criteria for recruitment at baseline including those without history of CHD. This study was approved by the Ethics Committee of the Mashhad University of Medical Sciences (MUMS) (MASHAD study code: 85134)^15^. All methods were performed in accordance with the relevant guidelines and regulations. Data on demographics, history of disease, smoking, depression and anxiety was collected using Beck and cardiovascular questionnaire at baseline.

### Follow up

We followed up individuals from 2011 to 2014 by phone. A total of 646 subjects were declared CHD events, so we invited them to the clinic to confirm CHD events. All patients were visited by cardiologist, and if needed, echocardiography assessment, cardiac scan and other examinations were done. Finally, cases with a possible CHD event were reviewed by a panel of specialists who agreed on a definite diagnosis. As a result of this follow-up, CHD events, including MI, stable angina and unstable angina, were confirmed in 219 subjects^16^.

### Anthropometric, biochemical data and nightly sleep

Systolic and diastolic blood pressure (SBP and DBP) were measured using a standard sphygmomanometer^17^. Fasting blood glucose, serum total cholesterol, LDL and high-density lipoprotein (HDL) cholesterol, triglycerides, uric acid and hs-CRP were measured as previously described^18^. The level of nightly sleep duration was assessed by questionnaire (self-reported), and based on the answer given to the question “how many hours do you sleep at night”, patients were categorized into four groups: < 5 h: very short nightly sleep, 5–6 h: short nightly sleep, 7–8 h: normal nightly sleep and > 9 h: long nightly sleep, in line with the majority of published studies^19,20^. Individuals who were night workers were determined^20^ by a self-questionnaire and excluded from the study.

### QRISK

QRISK is an estimation of the 10-year risk of CHD, and it was calculated with adjustments made as suggested by the Joint British Societies’ (JBS2) paper and the JBS Cardiovascular Risk Assessor (patient.info/doctor/cardiovascular-risk-calculator)^21,22^.

### Statistical analysis

Subjects in this study were divided into four groups according to nightly sleep: nightly sleep < 5 h, 5–6 h, 7–8 h and ≥ 9 h. Descriptive statistics, including the mean ± standard deviation, were considered for normally distributed variables or the median and interquartile range for variables that were not normally distributed. Differences in variables among nightly sleep were determined using ANOVA for normally distributed variables and Kruskal–Wallis H (the normality of distribution was evaluated using the Kolmogorov–Smirnov test). Chi square (or Fisher’s exact test) analysis was used for categorical parameters. Multinomial logistic regression was used to evaluate the odds ratio (OR) of event status and nightly sleep categorization. Relative risks (RRs) and 95% confidence intervals (CIs) of morbidity of all cases for nightly sleep were determined by using Cox’s proportional hazard model. All statistical analyses were undertaken by SPSS version 18 (SPSS Inc. Chicago, IL, USA). GraphPad Prism 6 and Adobe Illustrator CC | Graphic Design Software for figures were used. All the analyses were two-sided, and a p-value < 0.05 was considered significant.

## Results

### General characteristics of the subjects

Some characteristics of the subjects are shown in Table 1. Of the 9596 subjects in this study, 456 (4.8%) were found to have very short nightly sleep (< 5 h), 3065 (31.9%) had short nightly sleep (5–6 h), 5092 (53.1%) had normal nightly sleep (7–8 h) and 983 (10.2%) had long nightly sleep > 8 h). The mean ages of the groups were 51.27 ± 7.76, 48.99 ± 7.96, 47.33 ± 8 and 46.81 ± 8.2 years. The prevalence of short nightly sleep among the study population was 33.7% and 30.8% in men and women, respectively; and the prevalence of long nightly sleep was 8.2% and 11.6% in men and women, respectively. There were significant differences between men and women in short and long nightly sleep (p < 0.05) (Table 1).

**Table 1:** Demographic and biochemical characteristics of subjects according to nightly sleep.

		< 5 h (very short)	5–6 h (short)	7–8 h (normal)	> 8 h (long)	p-value
Total (9596)		456 (4.8%)	3065 (31.9%)	5092 (53.1%)	983 (10.2%)	
Age (y)		51.27 ± 7.76	48.99 ± 7.96^a^	47.33 ± 8.0^ab^	46.81 ± 8.2^ab^	< 0.001
Sex	Male	178 (4.6%)	1292(33.7%)	2056 (53.6%)	313 (8.2%)	< 0.001
	Female	278 (4.8%)	1773 (30.8%)	3036 (52.7%)	670 (11.6%)*	
Weight (kg)		71.49 ± 12.74	51.27 ± 7.76	51.27 ± 7.76	51.27 ± 7.76^bc^	< 0.001
Smoking	No	290 (4.4%)	2179 (33.1%)	3534 (53.7%)	574 (8.7%)	< 0.001
	Ex	52 (5.5%)	283 (29.9%)	505 (53.3%)	108 (11.4%)	
	Yes	114 (5.5%)	603 (29.1%)	1053 (50.8%)	301 (14.5%)*	
BMI (kg/m^2^)		28.27 ± 4.72	28.11 ± 4.77	27.83 ± 4.74^b^	27.57 ± 4.79^b^	0.003
SBP (mmHg)		122.73 ± 18.88	122.48 ± 17.91	121.12 ± 17.88^b^	119.98 ± 18.29^ab^	< 0.001
DBP (mmHg)		79.44 ± 11.02	79.44 ± 11.02	78.44 ± 11.02^b^	78.44 ± 11.02^b^	< 0.001
Glucose (mg/dl)		97.27 ± 47.99	93.1 ± 38.6	92.54 ± 39.23	92.54 ± 41.87	0.113
Uric acid (mg/dl)		4.91 ± 1.49	4.74 ± 1.38	4.65 ± 1.42^ab^	4.39 ± 1.27^abc^	< 0.001
Cholesterol (mg/dl)		194.09 ± 39.32	190.63 ± 39.25	192.48 ± 40.1	190.87 ± 38.55	0.111
hsCRP (mg/l)		1.93 (1.05–4.89)	1.66 (0.98–3.66)^a^	1.6 (0.99–3.43)^a^	1.64 (1.04–3.46)^a^	0.001
HDL (mg/dl)		42.83 ± 9.71	42.57 ± 10.26	42.9 ± 9.81	42.28 ± 9.51	0.254
TG (mg/dl)		122 (90–168)	118 (84–171.75)	121 (84–175)	123 (88–176)	0.36
LDL (mg/dl)		116.26 ± 36.36	115.65 ± 35.24	117.77 ± 35.81	117.10 ± 35.23	0.08
QRISK		12.81 ± 9.47	11.04 ± 9.01^a^	10.24 ± 8.92^ab^	9.97 ± 9.09^ab^	< 0.001
Event	No	428 (4.6%)	2994 (32%)	4977 (53.2%)	963 (10.3%)	< 0.001
	Yes	28 (5.1%)*	71 (30.3%)	11.5 (49.1%)	20 (8.5%)	

Data are presented as the mean (SD) or interquartile range. Differences in variables among nightly sleep determined using ANOVA analyses.

^a^< 5 h vs 5–6 h, 7–8 h and > 8 h.

^b^5–6 h vs 7–8 h and > 8 h; 7–8 h vs > 8 h.

The prevalence of no smoker, ex-smoker and current smoker in subjects with long nightly sleep were 8.7%, 11.4% and 14.5%, respectively. Current smoker subjects had significantly more nightly sleep > 8 h than other groups (p < 0.05). The mean weight, uric acid, hsCRP and QRISK of subjects with very short nightly sleep were significantly higher than those of the other groups (p < 0.05) (Table 1). The mean systolic and diastolic blood pressure and BMI were significantly lower in the groups with normal and long nightly sleep (p < 0.05).

### Prevalence of CHD events according to nightly sleep

**Figure 1:**
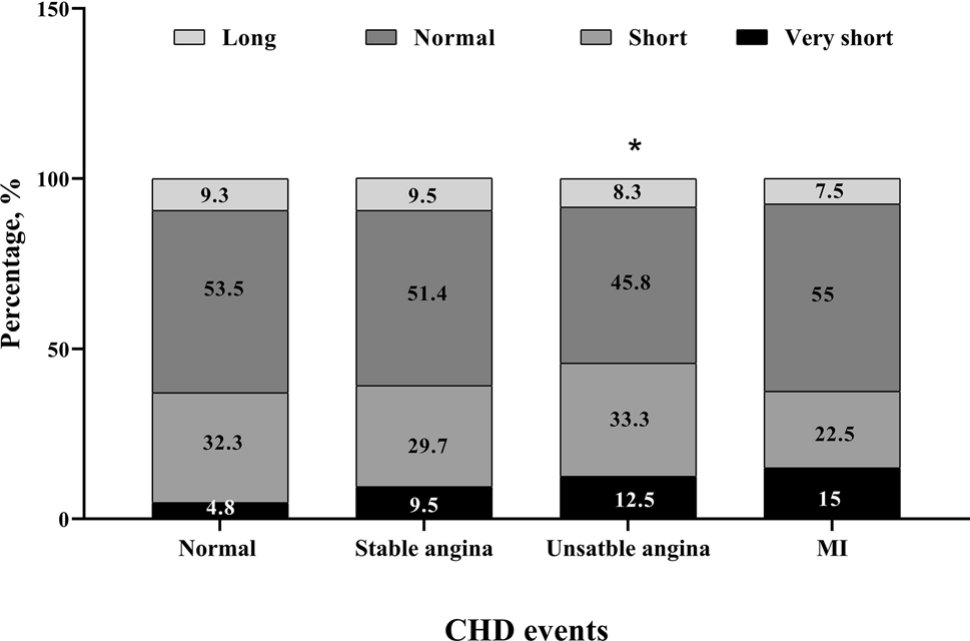
Prevalence of CHD events according to nightly sleep; *p < 0.05.

Of the individuals with stable angina, 9.5%, 29.7%, 51.4% and 9.5% had very short, short, normal and long nightly sleep, respectively. Incidence of CHD was significantly higher in participants with very short sleep duration than other groups. Of the group with unstable angina, 12.5%, 33.3%, 45.8% and 8.3% had very short, short, normal and long nightly sleep, respectively, and of the individuals with MI, 15%, 22.5%, 55% and 7.5% had very short, short, normal and long nightly sleep, respectively (Fig. 1). There were significant differences between very short nightly sleep with unstable and MI (p < 0.05).

### Nightly sleep indices as risk factors for CHD events

Multinomial logistic regression was performed to assess the odds ratio (OR) of the association between nightly sleep and CHD events. According to the data presented in Table 2, subjects with very short (< 5 h) nightly sleep had 2.831 OR of total event (CI 1.852–4.329), 3.241 OR of unstable angina (CI 1.772–5.925) and 2.525 OR of MI (CI 1.113–5.728). These data were recalculated after adjusting for sex, age, smoking, BMI, and systolic and diastolic blood pressure, and retained for total event and unstable angina (OR: 2.453 (CI 1.465–4.108)) (p < 0.05).

**Table 2:** Multinomial logistic regression of nightly sleep according to the event status.

	Unadjusted OR (CI 95%)	p-value	Adjusted OR (CI 95%)*	p-value
Total event				
Nightly sleep	Reference (Normal, 7–8 h)			
Very short (< 5 h)	2.831 (1.852–4.329)	** < 0.001**	2.176 (1.408–3.363)	** < 0.001**
Short (5–6 h)	1.026 (0.761–1.384)	0.865	0.872 (0.642–1.184)	0.37
Long (> 8 h)	0.899 (0.556–1.452)	0.663	0.973 (0.598–1.582)	0.911
Stable angina				
Nightly sleep	Reference (Normal, 7–8 h)			
Very short (< 5 h)	1.543 (0.602–3.953)	0.36	1.157 (0.522–2.256)	0.76
Short (5–6 h)	1.011 (0.594–1.722)	0.96	0.893 (0.57–1.4)	0.68
Long (> 8 h)	1.115 (0.495–2.515)	0.793	1.129 (0.568–2.241)	0.77
Unstable angina	Reference (Normal)			
Very short	3.241 (1.772–5.925)	** < 0.001**	2.453 (1.465–4.108)	**0.004**
Short	1.275 (0.829–1.963)	0.26	1.1 (0.764–1.585)	0.66
Long	0.956 (0.451–2.028)	0.9	1.031 (0.546–1.946)	0.93
MI	Reference (Normal)			
Very short	2.525 (1.113–5.728)	0.006	1.939 (0.845–4.452)	0.19
Short	0.602(0.305–1.188)	0.21	0.509 (0.257–1.008)	0.104
Long	0.521(0.154–1.759)	0.37	0.579 (0.171–1.963)	0.46

*Adjusted by sex, age, smoking, BMI, SBP and DBP.

Significant values are in bold.

**Figure 2:**
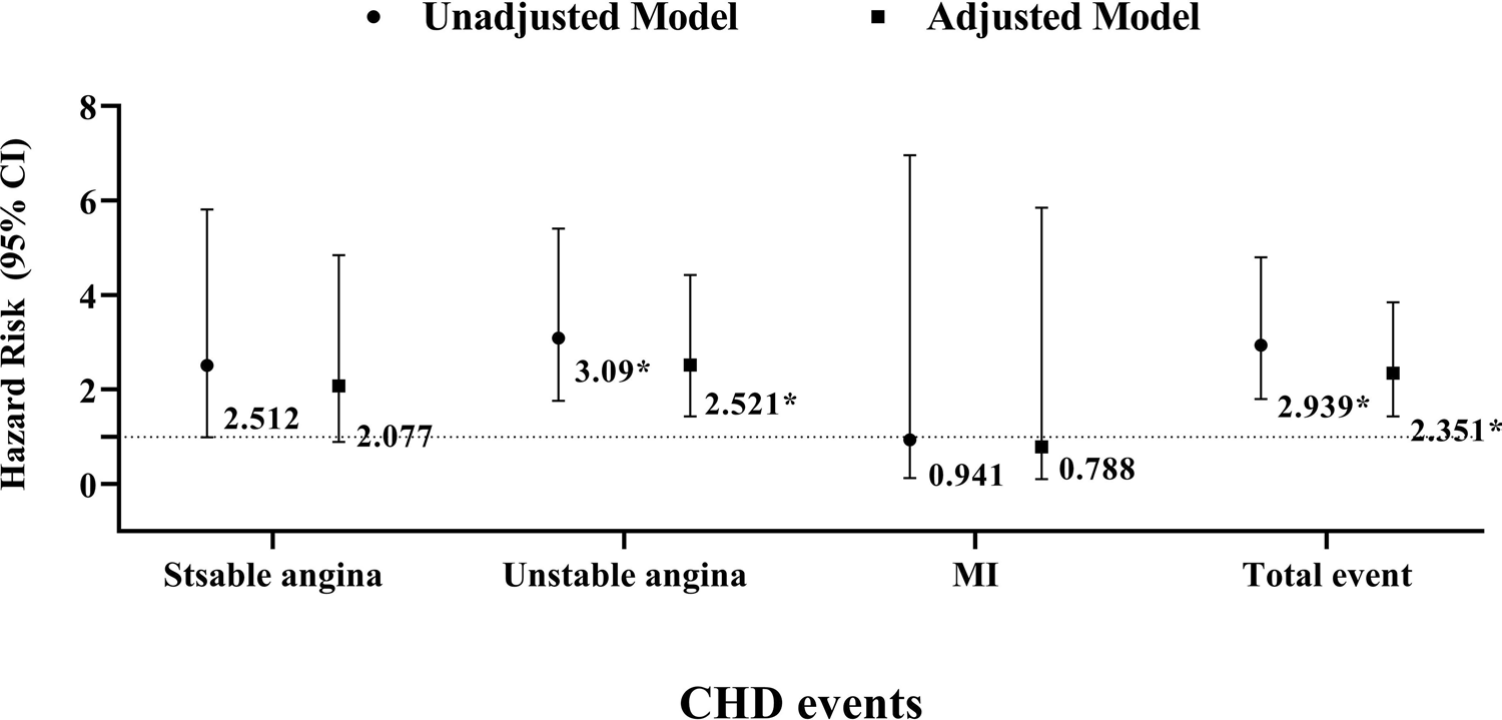
Cox regression model, CHD events and nightly sleep (≥ 5 h and < 5 h); *p < 0.05.

The relationship between CHD events and nightly sleep is presented in Fig. 2. According to this result, subjects with very short nightly sleep (< 5 h) had an increased relative risk for total event (RR: 2.939 (CI 1.878–4.770)) and unstable angina (RR: 2.614 (CI 1.354–5.047)) (p < 0.05). These results were confirmed after adjusting for sex, age, smoking, BMI, and systolic and diastolic blood pressure (Fig. 2). There were no significant differences between nightly sleep and the risk of stable angina and MI (Fig. 2).

**Figure 3:**
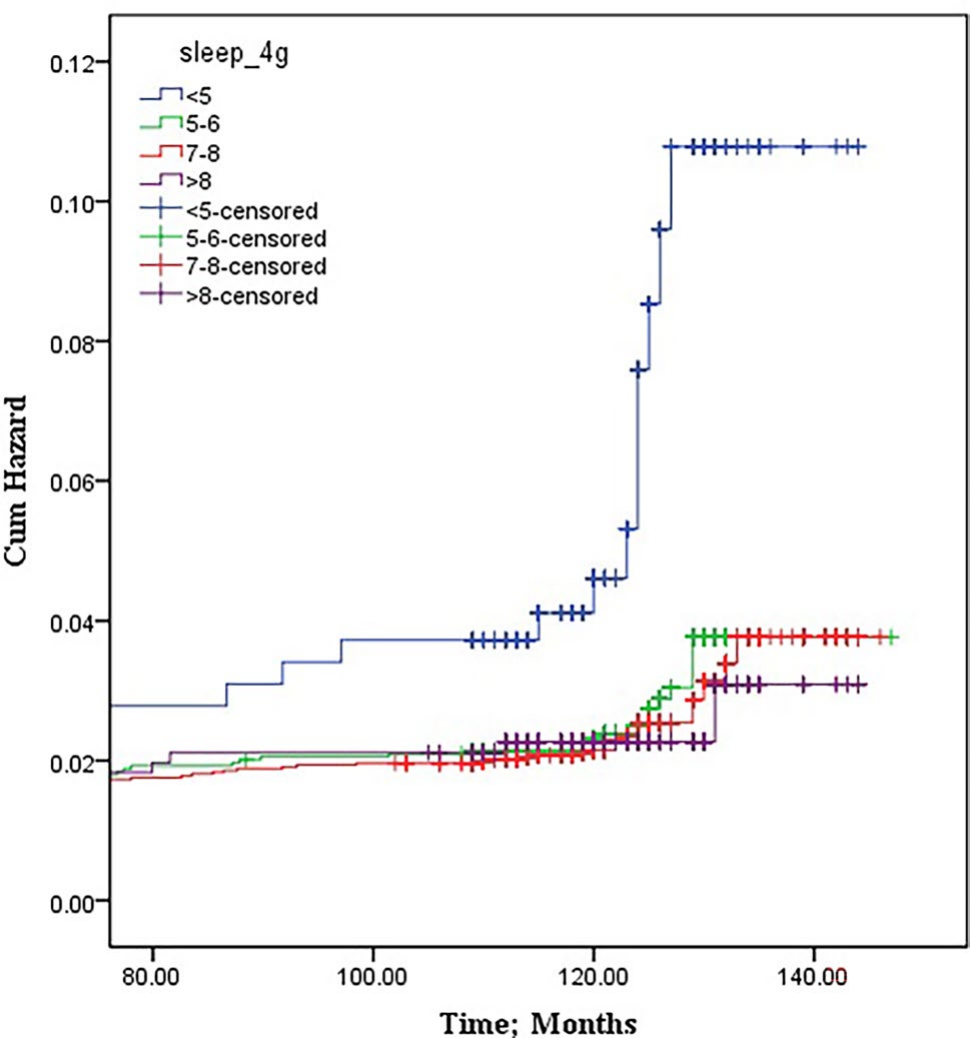
Survival Kaplan-Meyer curve, CHD events and nightly sleep.

Based on results shown in Table 3, there were 5 and 13 events at end of 108 and 120 months in nightly sleep < 5 h and hazard rate for these subjects was 0.002 and 0.01, respectively (the number of events and hazard rate for other groups are reported in Table 3). Also, for all the four groups, the survival during the follow up period is given in Fig. 3.

**Table 3:** Hazard rate, number at risk and number of event based on nightly sleep in study population.

Nightly sleep	Interval start time (years)	Number exposed to risk	Number of terminal events	Cumulative proportion surviving at end of interval	Hazard rate
< 5 h	8	3310.00	0	10.00	0.00
	9	2730.00	**5**	**0.98**	**0.002**
	10	122.50	**13**	**0.88**	**0.01**
	11	120.00	0	0.88	0.00
	12	10.00	0	0.88	0.00
5–6 h	8	24,260.00	0	10.00	0.00
	9	20,690.00	**13**	**0.99**	**0.00**
	10	9720.00	**38**	**0.95**	**0.00**
	11	112.50	**3**	**0.93**	**0.00**
	12	7.50	0	0.93	0.00
7–8 h	8	3982.50	0	10.00	0.00
	9	3389.50	**27**	**0.99**	**0.00**
	10	1609.50	**37**	**0.97**	**0.00**
	11	221.50	**7**	**0.94**	**0.00**
	12	140.00	0	0.94	0.00
> 8 h	8	721.50	0	10.00	0.00
	9	632.50	**5**	**0.99**	**0.00**
	10	308.50	**9**	**0.96**	**0.00**
	11	380.00	**1**	**0.94**	**0.00**
	12	3.50	0	0.94	0.00

Significant values are in bold.

## Discussion

Our project was the first study to investigate the association of sleep patterns with the incidence of coronary heart disease in a large cohort study in an Iranian population.

The results showed that there were significant differences between men and women in short and long nightly sleep. Current smoker subjects had significantly nightly sleep ≥ 9 h more than other groups. There were significant differences in mean weight, uric acid, hsCRP and QRISK between individuals with very short nightly sleep and other groups. The incidence of CHD was significantly higher in participants with very short sleep durations (< 5 h) than in other groups (p < 0.001). There were significant differences between very short nightly sleep and unstable and MI, and subjects with very short (< 5 h) nightly sleep had 3.241 OR of unstable angina (CI 1.772–5.925) and 2.525 OR of MI (CI 1.113–5.728). Subjects with very short nightly sleep (< 5 h) had an increased relative risk for unstable angina after adjusting for sex, age, smoking, BMI, and systolic and diastolic blood pressure.

Association of unhealthy sleep behaviors and poor sleep with survival and CVD outcomes and risk factors, has been previously reported^3,8,11,23^.

Grandner et al. concluded that inadequate sleep duration and sleep disturbance are related to obesity, dyslipidemia, diabetes, inflammation, high blood pressure and CVD. Therefore, it could be proposed that lack of or shortened sleep is a risk factor for cardiometabolic disability and fatality^8^.

In Taiwanese people, Chao et al. investigated association between length of sleep and prediabetes or newly diagnosed diabetes. Shortened of sleep could be a risk factor for the development of diabetes mellitus as well as sleep length were independently related to recently diagnosed diabetes^24^. Ferrie et al. revealed that an increase 2 h to night sleep associated with high incidence of diabetes in comparison to reference group with sufficient and proper sleep (7 h), odds ratio 1.65 [95% CI 1.15, 2.37] obtained with adjusted for age, gender, an occupation status, and ethnic categories. These results could be interpreted with weight gain in this group^25^. Kowall et al. also evaluated the role of sleep in incident prediabetes and type 2 diabetes mellitus as CVD risk factor in large population-based study. They demonstrated that there is a U-shaped correlation between sleep duration and incident diabetes also there isn’t any significant association between daily nap and incidence of it^17^.

Vozoris et al. found that sleep disorder symptoms accompanied with chronic short sleep time could be positively correlated to increased blood pressure, but the absence of relation with objective hypertension measures proposes that there might be no accurate association present^18^. Clark et al. examined significance of disturbed sleep on high blood pressure, diabetes mellitus and dyslipidemia as risk factors for CVD in 8 years follow up of the Finnish Public Sector study population. The results indicated that impaired sleep could be a predictor of hypertension and dyslipidemia with hazard ratio = 1.22, 95% CI 1.04–1.44 and HR = 1.17, 95% CI 1.07–1.29, respectively in completely adjusting^26^. Khan and Aouad examined some articles about sleep disorder, CVD and its risk factors like diabetes, high systolic blood pressure and cytokine concentrations. They expressed that people with normal blood pressure status suffered from chronic loss and shortened of sleep have shown increased or blunting systolic blood pressure (SBP) at night comparison to control group with normal sleep matched for age and gender^3,27^ as well as the rate of type 2 diabetes mellitus developed in subjects with sleep disorder^28^.

Spiegelhalder et al. in their systematic review, include of 10 studies, found that there were significant association between sleep disorder and coronary artery disease may be due to high blood pressure or elevated RHR. The risk ratios (RR) for CVD in individual with poor sleep was 1.5–3.9 when adjusted for some CVD risk factors such as smoking, hypertension, diabetes mellitus, age and fatness^4^.

In subjects of 385 292 UK Biobank study, the association of loss and shortened of sleep with genetic predisposition, and incidence of CVD have been investigated and their results indicated that appropriate and adequate sleep status have decreased risk of CVD between individual with low, intermediate, or high genetic risk^11^.

Li et al. assessed relationship among sleep disorder symptoms and fatality in US men population longitudinally during 6 years. Men with difficulty in beginning sleep and staying asleep, respectively had a 55% (HR 1.55; 95% CI1.19–2.04; p-trend = 0.01) and 32% (HR 1.32; 95% CI 1.02–1.72; p-trend = 0.002) high risk of CVD fatality, compared to men with normal sleep status^29^.

Bertisch and colleagues investigated the effect of chronic shortened of sleep on incident CVD and fatality in 4994 subjects of Sleep Heart Health Study (SHHS) well accomplished baseline polysomnography. Their findings showed that short sleep duration (< 5) related with an increased risk of CVD^30^.

Sofi et al. in their systematic review investigated the relationship of sleep disturbance with CVD in 122,501 individuals in 13 cohort studies. This population were followed 3 up to 20 years. They provided evidence that insomnia has led to an increased risk (‏45%) or mortality of CVD (RR, 1.45, 95% CI, 1.29–1.62; p < 0.00001)^5^.

Spiegelhalder et al. in their systematic review, include of 10 studies, found that there were significant association between sleep disorder and coronary artery disease may be due to high blood pressure or elevated RHR. The risk ratios (RR) for CVD in individual with poor sleep was 1.5–3.9 when adjusted for some CVD risk factors such as smoking, hypertension, diabetes mellitus, age and fatness^4^.

Li et al. in their meta-analysis of 17 cohort studies through 17 May 2014, showed that loss and shortened sleep were significantly related to the development risk of CVD and death after adjusting for classic CVD risk factors. They interpreted the association of insomnia with CVD through metabolic or endocrine modifications by increased concentrations of inflammatory cytokines^6^. Carreras et al., examined the effects of Sleep Fragmentation (SF) on function of endothelial and vessel wall construction in mice. They found that SF might lead to inflammation, and changes in structure of vessel and endothelial dysfunction^31^. Also, inadequate sleep duration might affect ghrelin and leptin level; this may lead to increased appetite and consequently obesity, impaired glycemic control, higher levels of cortisol, changes in growth hormone concentration, coronary artery calcification, and higher risk of atherosclerosis^13,23^.

Our results demonstrated that the incidence of CHD was significantly higher in participants with very short sleep durations than in other groups, and subjects with shortened sleep durations (< 5 h) had an increased relative risk for unstable angina after adjusting for several CVD risk factors. The results of the current study are largely consistent with the studies mentioned above and showed that inadequate sleep duration is associated with increased risk of CHD. Thus, we emphasize the importance of maintaining healthy/normal sleep pattern for prevention of CVD^1^.

## Conclusion

We found that chronic shortened sleep were associated with increased incident coronary heart disease in an Iranian population. There were significant differences between shortened nightly sleep with unstable and MI. Subjects with very short sleep had an increased relative risk for unstable angina after adjusting for several CHD risk factors. These findings suggest that sleep disorders, especially lack or shortened sleep, could be a risk factor for CHD.

## Limitation

This experiment should be re-conducted over a longer follow-up period for CHD events. Moreover, since we performed this research in MASHAD cohort study (i.e. on the participants recruited from Mashhad, eastern Iran), other populations of different genetic background and with different environmental exposures should be considered in future studies.

## Data Availability

The datasets generated and/or analyzed during the current study are not publicly available due copy right in Mashhad university of medical sciences but are available from the corresponding author on reasonable request.
